# Knowledge, Attitudes, and Perceptions Towards Male Circumcision Among Men Aged 20–40 Years in Otjiwarongo, Otjozondjupa Region, Namibia

**DOI:** 10.3390/ijerph23060808

**Published:** 2026-06-18

**Authors:** Masilu Daniel Masekameni, Joinet Sitapata, Phoka Caphus Rathebe, Themba Titus Sigudu

**Affiliations:** 1 Department of Development Studies, School of Social Sciences, University of South Africa, Pretoria 0003, South Africa; 2 Department of Environmental Health, Faculty of Health Sciences, University of Johannesburg, Johannesburg 2092, South Africa; jsitapata@gmail.com (J.S.); prathebe@uj.ac.za (P.C.R.); 3 Division of Health and Society, School of Public Health, Faculty of Health Sciences, University of the Witwatersrand, Johannesburg 2000, South Africa; themba.sigudu@wits.ac.za

**Keywords:** VMMC, HIV prevention, knowledge, attitudes, public health education

## Abstract

**Highlights:**

**Public health relevance—How does this work relate to a public health issue?**

- Male circumcision is a proven intervention for reducing HIV transmission, yet uptake remains suboptimal in non-circumcising communities, highlighting a critical public health gap.
- Understanding knowledge, attitudes, and perceptions towards VMMC is essential for addressing behavioural barriers to HIV prevention in high-burden settings.

**Public health significance—Why is this work of significance to public health?**

- The study identifies persistent knowledge gaps and misconceptions despite high awareness, which may limit the effectiveness of VMMC programmes.
- It demonstrates that education level, knowledge, and circumcision status are key determinants of favourable attitudes and perceptions, highlighting the importance of health literacy in HIV prevention.

**Public health implications—What are the key implications or messages for practitioners, policy makers and/or researchers in public health?**

- Targeted, culturally appropriate health education interventions are needed to address misconceptions and improve acceptance of VMMC, particularly among rural and less-educated populations.
- Strengthening community engagement strategies and integrating VMMC promotion within broader HIV prevention programmes can enhance uptake and contribute to population-level reductions in HIV transmission.

**Abstract:**

Male circumcision (MC) has been shown in studies from South Africa, Kenya, and Uganda to reduce heterosexual Human Immunodeficiency Virus (HIV) transmission among men by approximately 50–60%. Despite Namibia’s adoption of voluntary medical male circumcision (VMMC) as part of national HIV prevention strategies, uptake remains suboptimal in some communities. This study assessed the knowledge, attitudes, and perceptions (KAP) of male circumcision among men aged 20–40 years in Otjiwarongo, Namibia, and examined socio-demographic factors associated with these outcomes. A community-based cross-sectional survey was conducted between March and May 2024, involving 359 participants selected through multistage sampling. Data were collected using structured, pre-tested questionnaires and analysed using STATA version 19. Descriptive statistics, chi-square tests, and binary logistic regression were used to summarise data and identify predictors of favourable KAP outcomes. Overall, 69.1% of respondents demonstrated good knowledge of male circumcision, 72.7% had positive attitudes, and 69.9% reported positive perceptions. Awareness of male circumcision was high (91.9%); however, only 69.4% of participants recognised its role in reducing HIV infection risk, and notable misconceptions persisted regarding its protective effects and procedural aspects. Multivariable analysis showed that urban residence (AOR = 1.58; 95% CI: 1.03–2.42; *p* = 0.035) and higher education (AOR = 4.12; 95% CI: 1.21–14.02; *p* = 0.024) were significant predictors of favourable KAP outcomes. In addition, good knowledge was strongly associated with positive attitudes (AOR = 3.25; 95% CI: 2.01–5.26; *p* < 0.001) and perceptions (AOR = 2.87; 95% CI: 1.79–4.60; *p* < 0.001). In conclusion, while knowledge, attitudes, and perceptions towards male circumcision were generally favourable, important gaps and misconceptions remain. Targeted, culturally appropriate health education interventions are needed, particularly among rural and less-educated populations, to improve understanding and acceptance of VMMC as part of comprehensive HIV prevention strategies.

## 1. Introduction

Male circumcision (MC) has long been practiced for cultural, religious, and health reasons, but its medical significance gained renewed attention following randomized controlled trials in South Africa, Kenya, and Uganda demonstrating that MC can reduce heterosexual Human Immunodeficiency Virus (HIV) transmission among men by approximately 50–60% [1,2,3]. These findings led the World Health Organization (WHO) and the Joint United Nations Programme on HIV/AIDS (UNAIDS) to recommend integrating voluntary medical male circumcision (VMMC) into comprehensive HIV prevention strategies, particularly in high-prevalence and low-circumcision contexts [4]. In Namibia, the Ministry of Health and Social Services adopted VMMC as a national intervention to reduce HIV incidence [5]; however, uptake remains low in non-circumcising communities such as those in the Otjozondjupa Region. Research in southern Africa attributes this to cultural resistance, fear of pain, and misconceptions about post-circumcision sexual behaviour [6,7]. Additionally, disparities in education, residence, and socio-economic factors further influence acceptance patterns [8,9].

A growing body of literature highlights the importance of adequate knowledge in shaping MC uptake. Studies indicate that limited understanding of VMMC’s biomedical benefits, particularly the concept of partial HIV protection, can lead to low acceptance among eligible men [10]. When individuals incorrectly assume that MC offers full protection, they may either reject the procedure due to exaggerated expectations or adopt riskier sexual behaviour post-circumcision, such as reduced condom use, which undermines prevention efforts [11]. Furthermore, lack of awareness about the procedure’s safety, healing process, and availability through public health services often contributes to fear and scepticism, discouraging men from seeking circumcision [12].

Knowledge gaps also have broader public health consequences. Inaccurate beliefs about MC can affect community-level uptake, reducing the population-level protective effect necessary for significant declines in HIV transmission [4]. Misconceptions propagated within families and peer networks perpetuate misinformation, particularly in rural communities where formal health education is limited [13]. Evidence also shows that men with poor knowledge of HIV transmission pathways are less likely to adopt combined preventive measures such as consistent condom use and partner testing, which further accelerates HIV spread in high-burden settings [14].

Attitudes toward MC equally play a crucial role in determining whether men choose to undergo the procedure. Positive attitudes such as perceiving MC as enhancing hygiene, improving sexual satisfaction, or aligning with modern health practices have been shown to significantly increase uptake [15]. Conversely, negative attitudes stemming from cultural norms, fear of pain, and concerns about reduced masculinity remain major barriers, particularly in communities where circumcision is not traditionally practiced [16]. These attitudes are often reinforced by social expectations, peer influence, and family opinions, making them powerful determinants of behaviour.

Moreover, studies indicate that attitudes towards MC are shaped by perceptions of risk and trust in the health system. Men who view themselves as not at risk for HIV, or who rely heavily on traditional remedies, often express little interest in MC [17]. Distrust in healthcare providers, concerns about complications, and fears regarding abstinence during the healing period further discourage participation [18]. Attitudes can therefore both facilitate and hinder uptake, depending on whether they align with accurate information and supportive social norms.

Namibia continues to experience a significant HIV burden, with adult HIV prevalence estimated at approximately 11–12% among individuals aged 15–49 years [2]. As part of national HIV prevention strategies, voluntary medical male circumcision (VMMC) has been promoted as an effective biomedical intervention shown to reduce the risk of heterosexual HIV acquisition among men [3]. Understanding the knowledge, attitudes, and perceptions of men toward male circumcision in communities such as Otjiwarongo is therefore important for informing targeted HIV prevention programmes.

Despite the recognised role of knowledge and attitudes in influencing male circumcision uptake, there is limited empirical evidence on how these factors interact with socio-demographic characteristics among men in Otjiwarongo, Namibia, a setting where circumcision is not traditionally practiced. Understanding these relationships is important for designing targeted health promotion strategies and improving the effectiveness of voluntary medical male circumcision programmes.

Therefore, the primary research question of this study was: What are the levels of knowledge, attitudes, and perceptions regarding male circumcision among men aged 20–40 years in Otjiwarongo, Namibia, and how are these outcomes associated with socio-demographic characteristics? Addressing this question required examining the distribution of knowledge, attitudes, and perceptions within the study population and assessing their associations with factors such as age, residence, and educational attainment.

By identifying the determinants of knowledge and attitudes toward MC in this setting, the study provides evidence to support targeted health education interventions and culturally appropriate HIV prevention strategies, particularly those promoting VMMC as part of comprehensive HIV prevention efforts.

## 2. Materials and Methods

### 2.1. Study Design

This study employed a quantitative community-based cross-sectional design to assess knowledge, attitudes, and perceptions towards male circumcision among men aged 20–40 years in Otjiwarongo, Otjozondjupa Region, Namibia. A cross-sectional approach was appropriate because it enabled the assessment of population-level knowledge, attitudes, and perceptions at a single point in time and allowed examination of associations between socio-demographic characteristics and circumcision-related outcomes.

To address the study objectives, chi-square tests were used to examine associations between categorical variables, while binary logistic regression analysis was conducted to identify socio-demographic predictors of favourable knowledge, attitudes, and perceptions toward male circumcision.

### 2.2. Study Setting

**Figure 1:**
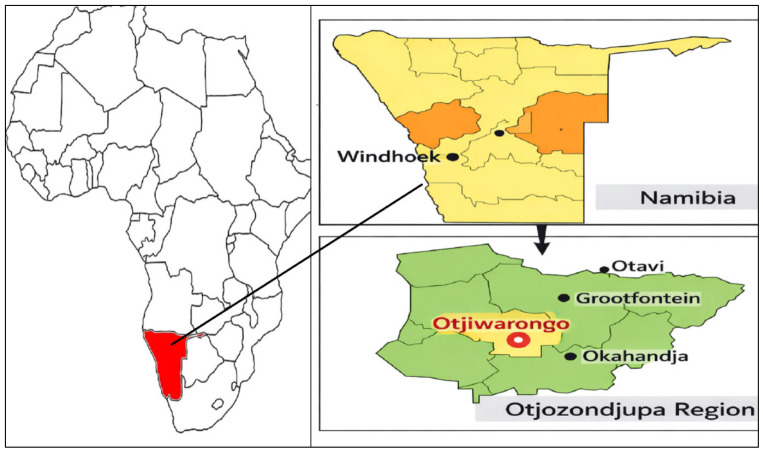
Geographical location of the study area showing Namibia within Africa and the position of Otjiwarongo in the Otjozondjupa Region (the red dot indicates the actual location of the study participants).

The study was conducted in Otjiwarongo, a town located in the Otjozondjupa Region in north-central Namibia (Figure 1). Otjiwarongo lies approximately 250 km north of Windhoek, the capital city of Namibia, and serves as the administrative and economic centre of the Otjozondjupa Region. The town has an estimated population of approximately 50,000 residents and functions as an important transportation and commercial hub linking northern, central, and southern Namibia through major road networks [1]. Due to its strategic location and diverse population drawn from surrounding rural and peri-urban communities, Otjiwarongo provides a relevant setting for examining public health behaviours and health-related knowledge among adult men.

### 2.3. Study Population

The study population comprised men aged 20–40 years residing in Otjiwarongo, located in the Otjozondjupa Region of Namibia, at the time of the survey. This age group was specifically selected because it represents a sexually active population at increased risk of HIV acquisition and is a key target group for voluntary medical male circumcision (VMMC) programmes.

### 2.4. Participants Recruitment

Participants were recruited from both urban and rural residential areas within Otjiwarongo to ensure representation of diverse socio-demographic characteristics, including variations in education level, cultural background, and access to health services. The study aimed to capture a broad cross-section of men from different communities to allow for meaningful assessment of knowledge, attitudes, and perceptions towards male circumcision, as well as associated socio-demographic determinants.

The inclusion of individuals who had resided in the area for at least six months ensured that participants were familiar with the local context, including available health services and community norms related to male circumcision. This was important for obtaining reliable responses regarding awareness, attitudes, and perceptions shaped by the local environment.

To enhance the representativeness of the study sample, a stratified sampling approach was employed. The study area was divided into relevant strata based on key socio-demographic characteristics, including residential location. Within each stratum, participants were selected proportionally to reflect the underlying population distribution. This approach ensured that individuals from different demographic backgrounds were adequately represented in the sample, thereby improving the generalisability of the findings to the broader population of men aged 20–40 years in Otjiwarongo.

### 2.5. Inclusion and Exclusion Criteria

Participants were eligible for inclusion in the study if they were male, aged between 20 and 40 years, had resided in Otjiwarongo for at least six months prior to the study, and were willing to provide informed consent to participate.

Individuals were excluded if they were younger than 20 years or older than 40 years, were temporary residents or visitors to Otjiwarongo, or declined to provide informed consent or were unable to participate in the interview process.

### 2.6. Sample Size Determination

The required sample size was calculated using the Yamane (1967) formula for finite populations with a 95% confidence level and a margin of error of 5%. Based on this calculation, a total sample size of 359 participants was determined to be sufficient to provide reliable estimates of knowledge, attitudes, and perceptions towards male circumcision in the study population.

A contingency percentage for non-response was not added to the calculated sample size, as the study employed an interviewer-administered questionnaire with direct household recruitment, which facilitated a high response rate and minimised the likelihood of non-response.

### 2.7. Sampling Procedure

A multistage probability sampling technique was used to recruit study participants. In the first stage, residential wards within Otjiwarongo were stratified according to population density and settlement type (urban and rural). In the second stage, households within selected wards were randomly selected using systematic sampling procedures. In the final stage, one eligible male respondent aged 20–40 years was randomly selected from each household using simple random sampling. This approach ensured that participants from different residential areas and socio-demographic backgrounds were represented in the study.

### 2.8. Data Collection

#### 2.8.1. Data Collection Instrument

Data were collected using a structured and pre-tested questionnaire administered through face-to-face interviews. The questionnaire comprised four main sections: the first collected socio-demographic information, including age, residence, religion, ethnicity, and education level; the second assessed knowledge about male circumcision, focusing on awareness of the procedure, understanding of its health benefits, and knowledge of its role in preventing HIV and other sexually transmitted infections; the third examined attitudes towards male circumcision, evaluating respondents’ beliefs regarding the benefits of circumcision, its cultural acceptability, and their willingness to recommend the procedure to others; and the fourth explored perceptions of male circumcision, including perceived health benefits, social acceptability, fears related to pain or complications, and beliefs regarding its effects on sexual performance. Knowledge questions were measured using binary response options (Yes/No), while attitude items were assessed using Likert-scale responses (agree, neutral, disagree).

Prior to the main data collection, the questionnaire was pilot-tested among respondents outside the study sample to assess clarity, cultural appropriateness, and reliability. The internal consistency of the instrument was evaluated using Cronbach’s alpha coefficient. The results indicated acceptable reliability for the overall questionnaire and its subscales, with Cronbach’s alpha values exceeding the recommended threshold of 0.70, demonstrating satisfactory internal consistency.

#### 2.8.2. Data Collection Procedure

Data were collected between March and May 2024 by trained field researchers fluent in English and relevant local languages. Interviews were conducted in private settings within households to ensure confidentiality and encourage honest responses. Fieldworkers received training on ethical conduct, informed consent procedures, questionnaire administration, and culturally sensitive engagement with participants. Completed questionnaires were reviewed daily by the research team to ensure completeness and accuracy.

### 2.9. Variable Measurement and Scoring

#### 2.9.1. Knowledge Score

Knowledge of male circumcision was assessed using six knowledge-related items. Each correct response was assigned a score of 1, while incorrect responses were scored 0, resulting in a total possible score ranging from 0 to 6.

The total knowledge score was categorised into good knowledge and poor knowledge based on a predefined cut-off point. Respondents who scored 4–6 points (≥50%) were classified as having good knowledge, while those who scored 0–3 points (<50%) were classified as having poor knowledge.

#### 2.9.2. Attitude Score

Attitudes towards male circumcision were assessed using a series of Likert-scale statements with response options of agree, neutral, and disagree. For analytical purposes, responses were dichotomised, where positive attitudes (agree) were assigned a score of 1, while neutral and negative responses (neutral and disagree) were assigned a score of 0. This approach was used to simplify interpretation and facilitate the construction of a composite attitude score.

The total attitude score was calculated by summing the individual item scores across all attitude-related questions, resulting in a cumulative score ranging from 0 to the total number of items included. Higher scores indicated more favourable attitudes towards male circumcision.

To enable categorical analysis, the total attitude score was further classified into positive attitude and negative attitude based on a predefined cut-off point. Respondents who scored ≥50% of the total possible score were classified as having a positive attitude, while those who scored <50% were classified as having a negative attitude.

The 50% cut-off threshold was selected based on its common application in cross-sectional KAP studies as a pragmatic and interpretable criterion for distinguishing between favourable and unfavourable attitudes in the absence of universally standardised scoring guidelines. This threshold reflects a minimum level at which respondents demonstrate generally positive orientations towards the subject under investigation.

The total attitude score was categorised into positive and negative attitude based on a predefined cut-off point. Respondents who scored 0.5–1 points (≥50%) were classified as having a positive attitude, while those who scored 0–0.49 points (≤50%) were classified as having a negative attitude.

#### 2.9.3. Perception Score

Perceptions about male circumcision were assessed using a series of binary response items related to hygiene, sexual performance, HIV prevention, social acceptability, and perceived barriers such as pain and complications. Each favourable perception was assigned a score of 1, while unfavourable perceptions were scored as 0, resulting in a total possible score ranging from 0 to the number of perception items included.

The total perception score was categorised into positive perception and negative perception based on a predefined cut-off point. Respondents who scored ≥50% of the total possible score were classified as having a positive perception, while those who scored <50% were classified as having a negative perception. The 50% cut-off was selected based on its common use in KAP studies as a pragmatic threshold to distinguish between adequate and inadequate knowledge in the absence of universally standardised criteria.

The total perception score was categorised into positive and negative perception based on a predefined cut-off point. Respondents who scored 0.5–1 points (≥50%) were classified as having a positive perception, while those who scored 0–0.49 points (≤50%) were classified as having a negative perception.

### 2.10. Data Analysis

Collected data were entered into Microsoft Excel and exported for analysis using STATA version 19 (StataCorp, College Station, TX, USA). Data cleaning procedures were conducted prior to analysis to check for completeness, consistency, and potential entry errors.

Descriptive statistics were used to summarise the socio-demographic characteristics of respondents as well as their knowledge, attitudes, and perceptions regarding male circumcision. Categorical variables were presented using frequencies, percentages, and 95% confidence intervals (CI). Summary measures were also used to describe key variables in the study population.

Knowledge, attitude, and perception responses were further analysed by constructing composite scores. Knowledge scores were derived from six knowledge-related items, where each correct response was assigned a score of 1 and incorrect responses a score of 0. The total scores were categorised into good knowledge and poor knowledge based on the overall distribution of scores. Attitude responses measured on Likert scales were grouped into positive attitudes and negative attitudes, while perception responses were categorised into positive perceptions and negative perceptions based on responses to perception-related items.

Bivariable analysis was performed to examine the association between socio-demographic characteristics and KAP outcomes. The Chi-square (χ^2^) test was used to assess statistical associations between categorical variables. Variables showing significant associations in the bivariable analysis were considered for inclusion in the multivariable model. In addition, variables of known epidemiological relevance (such as age, residence, education level, and circumcision status) were retained in the multivariable models irrespective of their bivariable significance to ensure appropriate control of potential confounding.

To identify independent predictors of knowledge, attitudes, and perceptions towards male circumcision, binary logistic regression analysis was conducted. Adjusted odds ratios (AORs) with corresponding 95% confidence intervals were calculated to estimate the strength of associations between explanatory variables and outcome variables while controlling for potential confounders. Statistical significance was determined at a *p*-value of less than 0.05.

### 2.11. Ethical Considerations

Ethical approval for the study was obtained from the University of Johannesburg Faculty of Health Sciences Research Ethics Committee (REC-2617-2024). Permission to conduct the study was also obtained from the Otjozondjupa Regional Health Authorities. In addition, approval to publish the findings was obtained from the Ministry of Health and Social Services (MOHSS), Namibia.

All participants provided written informed consent prior to participation. Participants were informed of the study objectives, the confidentiality of their responses, and their right to withdraw from the study at any time without penalty. All procedures were conducted in accordance with the principles of the Declaration of Helsinki.

## 3. Results

### 3.1. Socio-Demographic Characteristics

As shown in Table 1, a total of 359 respondents aged 20–40 years participated in the study, with all eligible individuals approached consenting to participate, yielding a response rate of 100%. The age distribution indicates that the largest proportion of respondents were 20–25 years old, accounting for 31.8% (*n* = 114; 95% CI: 27.0–36.6) of the sample. This was followed by participants aged 36–40 years, who constituted 27.3% (*n* = 98; 95% CI: 22.7–31.9). Respondents aged 31–35 years represented 21.2% (*n* = 76; 95% CI: 17.0–25.4) of the sample, while those aged 26–30 years comprised the smallest age group at 19.8% (*n* = 71; 95% CI: 15.7–23.9).

With regard to place of residence, the majority of respondents resided in rural areas, accounting for 62.4% (*n* = 224; 95% CI: 57.4–67.4) of the study population, while 37.6% (*n* = 135; 95% CI: 32.6–42.6) lived in urban areas.

In terms of religious affiliation, Christianity was the dominant religion among respondents, reported by 84.1% (*n* = 302; 95% CI: 80.3–87.9) of participants. A smaller proportion identified with traditional African religion, accounting for 3.9% (*n* = 14; 95% CI: 1.9–5.9), while 12.0% (*n* = 43; 95% CI: 8.6–15.4) reported belonging to other religious groups.

The ethnic composition of respondents was diverse, with the Ovambo ethnic group forming the largest proportion at 52.6% (*n* = 189; 95% CI: 47.4–57.8). This was followed by the Damara ethnic group, representing 18.9% (*n* = 68; 95% CI: 14.9–22.9) of the sample, and the Herero ethnic group, accounting for 11.4% (*n* = 41; 95% CI: 8.1–14.7). Participants from the San ethnic group comprised 7.5% (*n* = 27; 95% CI: 4.8–10.2), while 9.5% (*n* = 34; 95% CI: 6.4–12.6) reported belonging to other ethnic groups.

Regarding educational attainment, just over half of the respondents had completed secondary education, accounting for 51.8% (*n* = 186; 95% CI: 46.6–57.0) of the sample. A substantial proportion had primary education, representing 37.3% (*n* = 134; 95% CI: 32.3–42.3), while only 10.9% (*n* = 39; 95% CI: 7.7–14.1) reported having higher education.

The distribution of circumcision status among participants indicated that more than half of the respondents were uncircumcised, accounting for 52.1% (*n* = 187; 95% CI: 46.9–57.3), while 47.9% (*n* = 172; 95% CI: 42.7–53.1) reported having undergone circumcision.

### 3.2. Knowledge and Circumcision

As presented in Table 2, the majority of respondents demonstrated a high level of awareness of male circumcision. Overall, 91.9% (*n* = 330; 95% CI: 88.9–94.9) reported that they had heard about male circumcision, while only 8.1% (*n* = 29) indicated that they had never heard of the procedure.

Knowledge regarding the protective role of male circumcision against HIV infection was also relatively high. Approximately 69.1% (*n* = 248; 95% CI: 64.3–73.9) of respondents correctly reported that male circumcision reduces the risk of HIV transmission, whereas 30.9% (*n* = 111) did not recognise this protective effect. Similarly, 76.9% (*n* = 276; 95% CI: 72.5–81.3) of respondents indicated that male circumcision improves genital hygiene, while 23.1% (*n* = 83) disagreed with this statement.

Knowledge about the role of male circumcision in preventing sexually transmitted infections (STIs) was somewhat lower. About 61.0% (*n* = 219; 95% CI: 56.0–66.0) of participants reported that circumcision reduces the risk of STIs, whereas 39.0% (*n* = 140) were not aware of this benefit. Similarly, 61.6% (*n* = 221; 95% CI: 56.6–66.6) of respondents indicated that medical male circumcision is safer than traditional circumcision, while 38.4% (*n* = 138) did not perceive a difference in safety between the two procedures.

Knowledge regarding the appropriate age for circumcision was comparatively lower than other knowledge indicators. Slightly more than half of respondents, 56.5% (*n* = 203; 95% CI: 51.4–61.6), correctly identified the appropriate age for circumcision, whereas 43.5% (*n* = 156) were not aware of the recommended age.

### 3.3. Attitudes on Male Circumcision

As shown in Table 3, the majority of respondents expressed positive attitudes towards male circumcision. Most participants agreed that male circumcision is beneficial for men’s health, with 76.3% (*n* = 274) expressing agreement, while 11.4% (*n* = 41) were neutral and 12.3% (*n* = 44) disagreed with this statement.

Similarly, a substantial proportion of respondents supported the promotion of male circumcision. About 68.2% (*n* = 245) agreed that male circumcision should be promoted, whereas 15.3% (*n* = 55) remained neutral and 16.4% (*n* = 59) disagreed with promoting the practice.

Regarding willingness to advocate for the procedure, 72.7% (*n* = 261) of respondents indicated that they would recommend male circumcision to others, while 12.0% (*n* = 43) reported neutral views and 15.3% (*n* = 55) expressed disagreement.

Attitudes relating to cultural perceptions of male circumcision revealed that the majority of respondents did not perceive circumcision as conflicting with cultural beliefs. Specifically, 63.2% (*n* = 227) disagreed with the statement that circumcision conflicts with cultural beliefs, while 20.1% (*n* = 72) agreed and 16.7% (*n* = 60) were neutral.

Perceptions regarding the effect of circumcision on sexual satisfaction were more varied. Approximately 59.1% (*n* = 212) agreed that circumcision improves sexual satisfaction, while 19.2% (*n* = 69) were neutral and 21.7% (*n* = 78) disagreed with this statement.

### 3.4. Male Circumcision and Lived Expexperiences

As presented in Table 4, respondents demonstrated generally positive perceptions regarding male circumcision, although some concerns and misconceptions were also evident. A majority of participants, 69.4% (*n* = 249; 95% CI: 64.6–74.2), perceived that male circumcision reduces the risk of HIV infection, while 30.6% (*n* = 110) did not believe that circumcision provides such protection.

Similarly, 76.9% (*n* = 276; 95% CI: 72.5–81.3) of respondents believed that circumcised men are cleaner, indicating a strong perception of improved personal hygiene associated with circumcision. In contrast, 23.1% (*n* = 83) did not share this perception.

Perceptions regarding the impact of circumcision on sexual performance were more divided. Approximately 59.1% (*n* = 212; 95% CI: 54.0–64.2) of respondents believed that circumcision improves sexual performance, whereas 40.9% (*n* = 147) did not agree with this view.

In terms of social acceptance, about 66.3% (*n* = 238; 95% CI: 61.3–71.3) of participants considered male circumcision to be socially acceptable, while 33.7% (*n* = 121) reported that it was not socially acceptable.

Concerns related to the procedure were also reported. Approximately 39.3% (*n* = 141; 95% CI: 34.2–44.4) of respondents indicated that fear of pain discourages men from undergoing circumcision, whereas 60.7% (*n* = 218) did not perceive pain as a major deterrent. Similarly, 32.3% (*n* = 116; 95% CI: 27.5–37.1) believed that fear of complications discourages circumcision, while 67.7% (*n* = 243) did not consider complications to be a significant barrier.

### 3.5. Knowledge, Attitudes, and Perception Score Classification

As shown in Table 5, the overall classification of knowledge, attitudes, and perceptions towards male circumcision indicates generally favourable levels among the respondents. With regard to knowledge, the majority of participants demonstrated good knowledge, accounting for 69.1% (*n* = 248; 95% CI: 64.3–73.9) of the study population, while 30.9% (*n* = 111; 95% CI: 26.1–35.7) were classified as having poor knowledge about male circumcision.

Similarly, the distribution of attitude scores revealed that most respondents held positive attitudes towards male circumcision, representing 72.7% (*n* = 261; 95% CI: 68.0–77.4) of participants. In contrast, 27.3% (*n* = 98; 95% CI: 22.6–32.0) of respondents exhibited negative attitudes towards the practice.

A comparable pattern was observed for perception scores. Approximately 69.9% (*n* = 251; 95% CI: 65.2–74.6) of respondents were classified as having positive perceptions about male circumcision, whereas 30.1% (*n* = 108; 95% CI: 25.4–34.8) demonstrated negative perceptions.

### 3.6. Inferential Statistics

#### 3.6.1. Predictors of Good Knowledge of Male Circumcision

Table 6 presents the results of the logistic regression analysis examining factors associated with good knowledge of male circumcision.

In the univariate (crude) analysis, residence, education level, and circumcision status were significantly associated with good knowledge. Respondents residing in urban areas had higher odds of demonstrating good knowledge compared to those in rural areas (COR = 1.87; 95% CI: 1.18–2.97). Similarly, respondents with secondary education (COR = 1.85; 95% CI: 1.17–2.92) and higher education (COR = 8.62; 95% CI: 2.54–29.20) were more likely to have good knowledge compared to those with primary education. Circumcised participants also had significantly higher odds of good knowledge compared to uncircumcised participants (COR = 2.13; 95% CI: 1.34–3.38).

After adjusting for potential confounders, education level and circumcision status remained significant predictors of good knowledge. Respondents with higher education were more than four times as likely to have good knowledge compared to those with primary education (AOR = 4.28; 95% CI: 1.18–15.48; *p* = 0.027). In addition, circumcised participants were significantly more likely to demonstrate good knowledge than their uncircumcised counterparts (AOR = 1.79; 95% CI: 1.09–2.94; *p* = 0.022).

Although urban residence was associated with higher odds of good knowledge in the crude analysis, this association was no longer statistically significant after adjustment (AOR = 1.51; 95% CI: 0.93–2.45; *p* = 0.100). Similarly, secondary education showed a borderline association (AOR = 1.62; 95% CI: 0.98–2.69; *p* = 0.060).

Age group was not significantly associated with knowledge in either the crude or adjusted analyses (*p* > 0.05).

Overall, these findings indicate that higher educational attainment and circumcision status are independent predictors of good knowledge of male circumcision, while the effects of residence and age diminish after controlling for confounding variables.

#### 3.6.2. Predictors of Positive Attitudes Towards Male Circumcision

Table 7 presents the results of the logistic regression analysis examining factors associated with positive attitudes towards male circumcision.

In the univariate (crude) analysis, education level, knowledge score, and circumcision status were significantly associated with positive attitudes. Respondents with higher education had increased odds of having positive attitudes compared to those with primary education (COR = 2.88; 95% CI: 1.10–7.53). Similarly, participants with good knowledge were more likely to exhibit positive attitudes than those with poor knowledge (COR = 1.96; 95% CI: 1.21–3.18). Circumcised respondents also had significantly higher odds of positive attitudes compared to uncircumcised participants (COR = 2.54; 95% CI: 1.53–4.22).

After adjusting for potential confounders, knowledge score and circumcision status remained significant predictors of positive attitudes. Respondents with good knowledge were significantly more likely to have positive attitudes compared to those with poor knowledge (AOR = 1.78; 95% CI: 1.06–2.99; *p* = 0.029). In addition, circumcised participants were more than twice as likely to exhibit positive attitudes compared to uncircumcised participants (AOR = 2.11; 95% CI: 1.23–3.61; *p* = 0.007).

Although education level showed a significant association in the crude analysis, this association was no longer statistically significant after adjustment (secondary: AOR = 1.24; 95% CI: 0.76–2.04; *p* = 0.390; higher: AOR = 1.92; 95% CI: 0.70–5.25; *p* = 0.200). Similarly, residence and age group were not significantly associated with positive attitudes in either the crude or adjusted analyses (*p* > 0.05).

Overall, these findings indicate that good knowledge and circumcision status are independent predictors of positive attitudes towards male circumcision, highlighting the important role of awareness and personal experience in shaping attitudes.

#### 3.6.3. Predictors of Positive Perceptions Towards Male Circumcision

Table 8 presents the results of the logistic regression analysis examining factors associated with positive perceptions towards male circumcision.

In the univariate (crude) analysis, education level, knowledge score, and circumcision status were significantly associated with positive perceptions. Respondents with secondary education (COR = 1.82; 95% CI: 1.15–2.88) and higher education (COR = 2.46; 95% CI: 1.01–5.99) were more likely to report positive perceptions compared to those with primary education. Similarly, participants with good knowledge had significantly higher odds of positive perceptions compared to those with poor knowledge (COR = 2.12; 95% CI: 1.32–3.39). Circumcised respondents were also more likely to report positive perceptions compared to uncircumcised participants (COR = 1.99; 95% CI: 1.23–3.21).

After adjusting for potential confounders, knowledge score and circumcision status remained significant predictors of positive perceptions. Respondents with good knowledge were significantly more likely to have positive perceptions compared to those with poor knowledge (AOR = 1.72; 95% CI: 1.03–2.87; *p* = 0.038). Likewise, circumcised participants had significantly higher odds of positive perceptions compared to uncircumcised participants (AOR = 1.76; 95% CI: 1.06–2.92; *p* = 0.029).

Although education level was significantly associated with perceptions in the crude analysis, this association was no longer statistically significant after adjustment (secondary: AOR = 1.41; 95% CI: 0.87–2.30; *p* = 0.160; higher: AOR = 1.68; 95% CI: 0.67–4.20; *p* = 0.270). Similarly, residence and age group were not significantly associated with perceptions in either the crude or adjusted analyses (*p* > 0.05).

Overall, these findings indicate that good knowledge and circumcision status are independent predictors of positive perceptions towards male circumcision, while the effects of socio-demographic factors such as age, residence, and education diminish after controlling for confounding variables.

## 4. Discussion

This study assessed the knowledge, attitudes, and perceptions (KAP) towards male circumcision (MC) among men aged 20–40 years in Otjiwarongo, Namibia, and examined socio-demographic factors associated with these outcomes. Overall, the findings indicate relatively favourable levels of knowledge, attitudes, and perceptions towards MC. However, important knowledge gaps and socio-demographic disparities remain, which may influence the uptake of voluntary medical male circumcision (VMMC) in this setting [19,20,21].

The socio-demographic profile of the study participants was broadly consistent with available census and regional population data for Otjiwarongo and the Otjozondjupa Region, particularly with respect to the distribution of religion, ethnic group, and education level. The predominance of Christianity and the representation of major ethnic groups such as Ovambo, Damara, and Herero align with known demographic patterns in the region [22]. Similarly, the observed distribution of education levels reflects national trends reported in Namibia.

The high level of awareness of male circumcision (91.9%) observed in this study is consistent with findings from other southern African countries where VMMC has been widely promoted as part of HIV prevention programmes [23]. Public health campaigns and community outreach initiatives have likely contributed to this high awareness [24]. However, awareness did not necessarily translate into comprehensive knowledge, as approximately one-third of respondents were unaware of the protective effect of MC against HIV, and a substantial proportion lacked knowledge regarding its role in preventing sexually transmitted infections and the recommended age for circumcision. These findings suggest that while general awareness is widespread, detailed and accurate knowledge remains insufficient, which may limit informed decision-making regarding circumcision [25,26].

The proportion of respondents demonstrating good knowledge (69.1%) is comparable to studies conducted in similar settings, although the persistence of misconceptions is concerning [27,28]. Misunderstandings regarding the extent of HIV protection provided by MC may lead to either underutilisation of VMMC services or engagement in risky sexual behaviours due to a false sense of complete protection [29]. This highlights the importance of emphasising that MC provides partial protection and should be integrated with other preventive measures such as condom use and HIV testing [30].

Attitudes towards male circumcision were generally positive, with more than two-thirds of respondents supporting its promotion and indicating willingness to recommend the procedure. These findings are encouraging, as positive attitudes are a key determinant of behavioural uptake [31]. The perception that MC improves hygiene and contributes to men’s health likely underpins these favourable attitudes. Additionally, the finding that a majority of respondents did not perceive MC as conflicting with cultural beliefs suggests a potential shift in social norms, even in traditionally non-circumcising communities [32,33].

Despite these positive attitudes, some concerns remain, particularly regarding pain, complications, and sexual performance. Approximately one-third of respondents reported fear of pain or complications as potential barriers, indicating that procedural concerns continue to influence perceptions [34]. These findings align with previous studies that identify fear and misconceptions as significant barriers to VMMC uptake [35]. Addressing these concerns through targeted health education and counselling is therefore critical to improving acceptance [36].

Perceptions towards MC were also largely positive, with the majority of respondents recognising its role in reducing HIV risk and improving hygiene. However, perceptions regarding sexual performance and social acceptability were more varied, reflecting ongoing uncertainty and cultural influences [37]. The persistence of mixed perceptions suggests that community-level beliefs and social narratives continue to shape individual decision-making, highlighting the need for culturally sensitive interventions [38].

The multivariable analysis provides important insights into the determinants of KAP outcomes. Educational attainment emerged as a strong predictor, with individuals with higher education significantly more likely to demonstrate favourable knowledge, attitudes, and perceptions. This finding underscores the role of education in enhancing health literacy and the ability to interpret health information [39]. Similarly, urban residence was associated with better KAP outcomes, likely reflecting greater access to health services, information, and exposure to public health campaigns [40].

Notably, knowledge was strongly associated with both attitudes and perceptions. Respondents with good knowledge were significantly more likely to exhibit positive attitudes (AOR = 3.25; *p* < 0.001) and perceptions (AOR = 2.87; *p* < 0.001) towards MC. This finding highlight knowledge as a central driver of acceptance and supports the notion that improving knowledge can lead to more favourable attitudes and perceptions, ultimately influencing behaviour [41].

Circumcision status was also an important predictor, with circumcised individuals more likely to have good knowledge, positive attitudes, and positive perceptions. This may reflect experiential knowledge and increased exposure to health information during the circumcision process. It also suggests that individuals who have undergone circumcision may act as advocates within their communities, potentially influencing others’ attitudes and behaviours [42].

Age, religion, and ethnicity were not significant predictors in the adjusted models, suggesting that socio-cultural differences may be less influential than previously assumed when controlling for other factors such as education and knowledge. Our findings are in agreement with a separate study carried out in Kenya [43]. This finding indicates that interventions aimed at improving knowledge and access to information may have broad applicability across different demographic groups. However, in a comparative study carried out in Eastern and Southern Africa, the authors state that age, religion, and ethnicity were significant predictors of male circumcision uptake, with older age, being Muslim, and specific ethnicities consistently associated with higher circumcision rates across multiple countries in the study [44].

The findings of this study have important public health implications. Although knowledge and attitudes towards MC are generally favourable, the persistence of misconceptions and socio-demographic disparities highlights the need for targeted interventions. Health education programmes should focus on improving understanding of the partial protective effect of MC, addressing fears related to pain and complications, and promoting the integration of MC with other HIV prevention strategies. Special attention should be given to rural populations and individuals with lower levels of education, who were found to have less favourable KAP outcomes.

Furthermore, community-based approaches that engage local leaders, peer educators, and circumcised men as advocates may help to address cultural barriers and improve social acceptance. Strengthening the delivery of accurate, culturally appropriate information through both healthcare facilities and community platforms is essential for increasing VMMC uptake and achieving population-level reductions in HIV transmission [42].

This study has some limitations that should be considered when interpreting the findings. The cross-sectional design limits the ability to establish causal relationships between variables. Additionally, the use of self-reported data may introduce social desirability bias, particularly for sensitive topics such as sexual health and circumcision.

Furthermore, the selection of variables for multivariable analysis was primarily based on bivariable significance, which may have resulted in the exclusion of potentially important confounders. Although key variables were retained based on theoretical relevance, this approach may still introduce residual confounding.

Additionally, the use of a 50% cut-off to categorise knowledge, attitudes, and perceptions may be considered arbitrary and could influence the classification of outcomes. Although this threshold is commonly applied in KAP studies for interpretability, alternative cut-off points or scoring approaches may yield different distributions of results.

Despite these limitations, the study provides valuable insights into the factors influencing knowledge, attitudes, and perceptions towards MC in a non-circumcising community in Namibia. The use of a stratified sampling approach further strengthened the representativeness of the sample by ensuring proportional inclusion of participants across key demographic strata. Collectively, these factors suggest that the study findings are reasonably generalisable to the target population, although minor deviations from population distributions cannot be entirely excluded.

In conclusion, while the study demonstrates generally favourable knowledge, attitudes, and perceptions towards male circumcision among men in Otjiwarongo, important gaps and disparities remain. Educational attainment, urban residence, and knowledge levels were key determinants of favourable outcomes. Strengthening targeted, culturally appropriate health education interventions is essential to address misconceptions, improve acceptance, and enhance the uptake of VMMC as part of comprehensive HIV prevention strategies.

## 5. Conclusions

This study found that men aged 20–40 years in Otjiwarongo, Namibia, generally demonstrated favourable knowledge, attitudes, and perceptions towards male circumcision. Most respondents had heard of male circumcision, and substantial proportions recognised its benefits for HIV prevention, genital hygiene, and men’s health. Positive attitudes towards promoting and recommending male circumcision were also common, and overall perceptions were largely supportive. However, important misconceptions persisted, particularly regarding the extent of HIV protection, the prevention of sexually transmitted infections, and concerns related to pain, complications, and sexual performance.

The findings further showed that higher educational attainment, urban residence, good knowledge, and circumcision status were important predictors of favourable outcomes. In particular, knowledge emerged as a key factor shaping both attitudes and perceptions, underscoring the importance of accurate health information in influencing acceptance of male circumcision.

These results suggest that strengthening targeted and culturally sensitive health education interventions is essential to address persistent misconceptions and improve uptake of voluntary medical male circumcision in Otjiwarongo and similar non-circumcising communities. Efforts should particularly focus on rural populations and men with lower educational attainment, while reinforcing that male circumcision provides partial protection and should be promoted as part of a comprehensive HIV prevention package. Ultimately, improving community understanding and acceptance of male circumcision may contribute to increased uptake of VMMC and support broader HIV prevention goals in Namibia.

## Figures and Tables

**Table 1:** Socio-demographic characteristics of respondents.

Variable	Category	*n* ^1^	% ^2^	95% CI ^3^
Age group (yrs)	20–25	114	31.8	27.0–36.6
	26–30	71	19.8	15.7–23.9
	31–35	76	21.2	17.0–25.4
	36–40	98	27.3	22.7–31.9
Residence	Rural	224	62.4	57.4–67.4
	Urban	135	37.6	32.6–42.6
Religion	Christianity	302	84.1	80.3–87.9
	Traditional African religion	14	3.9	1.9–5.9
	Other	43	12.0	8.6–15.4
Ethnic group	Ovambo	189	52.6	47.4–57.8
	Damara	68	18.9	14.9–22.9
	Herero	41	11.4	8.1–14.7
	San	27	7.5	4.8–10.2
	Other	34	9.5	6.4–12.6
Education level	Primary	134	37.3	32.3–42.3
	Secondary	186	51.8	46.6–57.0
	Higher	39	10.9	7.7–14.1
Circumcision status	Circumcised	172	47.9	42.7–53.1
	Uncircumcised	187	52.1	46.9–57.3

^1^ *n*: Frequency, ^2^ %: Proportion, ^3^ 95% CI: 95% confidence interval.

**Table 2:** Knowledge about male circumcision.

Knowledge Variable	Yes		No		95% CI ^3^
	*n* ^1^	% ^2^	*n* ^1^	%	
Ever heard of male circumcision	330	91.9	29	8.1	88.9–94.9
Male circumcision reduces HIV risk	248	69.1	111	30.9	64.3–73.9
Circumcision improves genital hygiene	276	76.9	83	23.1	72.5–81.3
Circumcision reduces STIs	219	61.0	140	39.0	56.0–66.0
Medical circumcision safer than traditional	221	61.6	138	38.4	56.6–66.6
Correct age for circumcision	203	56.5	156	43.5	51.4–61.6

^1^ *n*: Frequency, ^2^ %: Proportion, ^3^ 95% CI: 95% confidence interval for the “Yes” response.

**Table 3:** Attitudes towards male circumcision.

Attitude Statement	Agree		Neutral		Disagree	
	*n* ^1^	% ^2^	*n* ^1^	% ^2^	*n* ^1^	% ^2^
Male circumcision is beneficial for men’s health	274	76.3	41	11.4	44	12.3
Male circumcision should be promoted	245	68.2	55	15.3	59	16.4
I would recommend male circumcision	261	72.7	43	12.0	55	15.3
Circumcision conflicts with cultural beliefs	72	20.1	60	16.7	227	63.2
Circumcision improves sexual satisfaction	212	59.1	69	19.2	78	21.7

^1^ *n*: Frequency, ^2^ %: Proportion.

**Table 4:** Perceptions about male circumcision.

Perception Statement	Yes		No		95% CI ^3^
	*n* ^1^	% ^2^	*n* ^1^	*n* ^1^	
Circumcision reduces HIV infection risk	249	69.4	110	30.6	64.6–74.2
Circumcised men are cleaner	276	76.9	83	23.1	72.5–81.3
Circumcision improves sexual performance	212	59.1	147	40.9	54.0–64.2
Circumcision socially acceptable	238	66.3	121	33.7	61.3–71.3
Fear of pain discourages circumcision	141	39.3	218	60.7	34.2–44.4
Fear of complications discourages circumcision	116	32.3	243	67.7	27.5–37.1

^1^ *n*: Frequency, ^2^ %: Proportion, ^3^ 95% CI: 95% confidence interval for the “Yes” response.

**Table 5:** Knowledge, attitudes and perception on male circumcision.

Variable	Category	Frequency (*n*)	Proportion (%)	95% CI ^1^
Knowledge score	Good knowledge	248	69.1	64.3–73.9
	Poor knowledge	111	30.9	26.1–35.7
Attitude score	Positive attitude	261	72.7	68.0–77.4
	Negative attitude	98	27.3	22.6–32.0
Perception score	Positive perception	251	69.9	65.2–74.6
	Negative perception	108	30.1	25.4–34.8

^1^ 95% CI: 95% confidence interval.

**Table 6:** Logistic regression analysis of predictors of good knowledge of male circumcision.

Variable	Category	Good Knowledge (*n*)	Poor Knowledge (*n*)	COR	95% CI	AOR	95% CI	*p*-Value
Age group (yrs)	20–25	76	38	0.78	0.47–1.29	0.82	0.48–1.41	0.470
	26–30	45	26	0.82	0.46–1.45	0.86	0.48–1.55	0.620
	31–35	53	23	0.95	0.52–1.72	0.97	0.53–1.79	0.930
	36–40	74	24	1	Reference	1	Reference	—
Residence	Rural	144	80	1	Reference	1	Reference	—
	Urban	104	31	1.87	1.18–2.97	1.51	0.93–2.45	0.100
Religion	Christianity	210	92	1	Reference	1	Reference	—
	Traditional	8	6	0.58	0.19–1.74	0.62	0.20–1.95	0.410
	Other	30	13	1.01	0.49–2.09	1.05	0.50–2.23	0.890
Ethnic group	Ovambo	135	54	1	Reference	1	Reference	—
	Damara	44	24	0.73	0.41–1.30	0.81	0.44–1.50	0.500
	Herero	28	13	0.86	0.41–1.80	0.92	0.42–2.01	0.840
	San	16	11	0.58	0.25–1.34	0.63	0.26–1.51	0.300
	Other	25	9	1.11	0.49–2.50	1.20	0.51–2.81	0.680
Education level	Primary	78	56	1	Reference	1	Reference	—
	Secondary	134	52	1.85	1.17–2.92	1.62	0.98–2.69	0.060
	Higher	36	3	8.62	2.54–29.20	4.28	1.18–15.48	0.027
Circumcision status	Uncircumcised	115	72	1	Reference	1	Reference	—
	Circumcised	133	39	2.13	1.34–3.38	1.79	1.09–2.94	0.022

Note: Adjusted for age group, residence, religion, ethnic group, education level, and circumcision status. COR: Crude Odds Ratio; AOR: Adjusted Odds Ratio; CI: Confidence Interval; Reference category indicated as “1”, the reference category for the *p*-value is represented using “—“.

**Table 7:** Logistic regression analysis of predictors of positive attitudes towards male circumcision.

Variable	Category	Positive Attitude (*n*)	Negative Attitude (*n*)	COR	95% CI	AOR	95% CI	*p*-Value
Age group (yrs)	20–25	78	36	0.84	0.50–1.41	0.88	0.51–1.52	0.640
	26–30	50	21	0.95	0.52–1.72	0.95	0.52–1.75	0.880
	31–35	56	20	1.05	0.57–1.93	1.06	0.57–1.99	0.850
	36–40	77	21	1	Reference	1	Reference	—
Residence	Rural	158	66	1	Reference	1	Reference	—
	Urban	103	32	1.35	0.83–2.20	1.18	0.71–1.97	0.520
Religion	Christianity	220	82	1	Reference	1	Reference	—
	Traditional	9	5	0.67	0.21–2.11	0.70	0.22–2.30	0.560
	Other	32	11	1.08	0.51–2.30	1.12	0.51–2.45	0.780
Ethnic group	Ovambo	140	49	1	Reference	1	Reference	—
	Damara	50	18	0.97	0.52–1.79	1.02	0.53–1.95	0.950
	Herero	30	11	0.96	0.45–2.05	1.00	0.45–2.22	0.990
	San	18	9	0.70	0.30–1.62	0.74	0.31–1.76	0.490
	Other	23	11	0.73	0.33–1.64	0.80	0.34–1.88	0.600
Education level	Primary	88	46	1	Reference	1	Reference	—
	Secondary	140	46	1.59	1.00–2.52	1.24	0.76–2.04	0.390
	Higher	33	6	2.88	1.10–7.53	1.92	0.70–5.25	0.200
Circumcision status	Uncircumcised	120	67	1	Reference	1	Reference	—
	Circumcised	141	31	2.54	1.53–4.22	2.11	1.23–3.61	0.007

Note: Adjusted for age group, residence, religion, ethnic group, education level, and circumcision status. COR: Crude Odds Ratio; AOR: Adjusted Odds Ratio; CI: Confidence Interval; Reference category indicated as “1” the reference category for the *p*-value is represented using “—“.

**Table 8:** Logistic regression analysis of predictors of positive perceptions towards male circumcision.

Variable	Category	Positive Perception (*n*)	Negative Perception (*n*)	COR	95% CI	AOR	95% CI	*p*-Value
Age group (yrs)	20–25	74	40	0.86	0.52–1.43	0.90	0.53–1.53	0.690
	26–30	48	23	0.93	0.52–1.67	0.96	0.53–1.76	0.900
	31–35	54	22	1.07	0.59–1.93	1.08	0.58–2.01	0.810
	36–40	75	23	1	Reference	1	Reference	—
Residence	Rural	151	73	1	Reference	1	Reference	—
	Urban	100	35	1.38	0.86–2.21	1.21	0.74–1.98	0.440
Religion	Christianity	212	90	1	Reference	1	Reference	—
	Traditional	8	6	0.57	0.18–1.73	0.60	0.19–1.90	0.380
	Other	31	12	0.90	0.44–1.84	0.94	0.44–2.01	0.880
Ethnic group	Ovambo	138	51	1	Reference	1	Reference	—
	Damara	48	20	0.89	0.49–1.63	0.95	0.51–1.78	0.880
	Herero	29	12	0.89	0.42–1.88	0.94	0.43–2.05	0.870
	San	17	10	0.63	0.27–1.47	0.68	0.28–1.66	0.400
	Other	19	15	0.47	0.22–1.02	0.52	0.23–1.18	0.120
Education level	Primary	82	52	1	Reference	1	Reference	—
	Secondary	138	48	1.82	1.15–2.88	1.41	0.87–2.30	0.160
	Higher	31	8	2.46	1.01–5.99	1.68	0.67–4.20	0.270
Circumcision status	Uncircumcised	118	69	1	Reference	1	Reference	—
	Circumcised	133	39	1.99	1.23–3.21	1.76	1.06–2.92	0.029

Note: Adjusted for age group, residence, religion, ethnic group, education level, and circumcision status. COR: Crude Odds Ratio; AOR: Adjusted Odds Ratio; CI: Confidence Interval; Reference category indicated as “1” the reference category for the *p*-value is represented using “—“.

## Data Availability

The data supporting the findings of this study are available from the corresponding author upon reasonable request.

## Abbreviations

The following abbreviations are used in this manuscript:

HIV	Human Immunodeficiency Virus
MC	Male Circumcision
MoHSS	Ministry of Health and Social Services
NGO	Non-Governmental Organization
STIs	Sexually Transmitted Infections
UNAIDS	Joint United Nations Programme on HIV/AIDS
VMMC	Voluntary Medical Male Circumcision
WHO	World Health Organization
